# Medical technologists’ experiences handling medical specimens during the COVID-19 pandemic

**DOI:** 10.4102/hsag.v30i0.2929

**Published:** 2025-07-04

**Authors:** Mamodiege C. Mafolo, Eunice Mutava, Alethea Sunnasy, Alida S. du Plessis-Faurie

**Affiliations:** 1 Department of Nursing, Faculty of Health Sciences, University of Johannesburg, Johannesburg, South Africa; 2 Aurum Institute, Johannesburg, South Africa

**Keywords:** South Africa, medical specimen, medical technologists, occupational exposure, SARS-CoV-2 virus, COVID-19 pandemic

## Abstract

**Background:**

The severe acute respiratory syndrome coronavirus type 2 virus (SARS-CoV-2) was highly infectious and had a high mortality rate. Globally, countries prioritised early disease detection through biological sample collection, analysis, and treatment. Understanding the physical and mental health outcomes that medical technologists experienced as a result of the increased risk of exposure is critical for making recommendations for the successful implementation of new workplace safety standards.

**Aim:**

This study attempts to provide a better understanding of medical technologists’ experiences and occupational exposure during the coronavirus disease 2019 (COVID-19) epidemic.

**Setting:**

The study was carried out at a Gauteng public laboratory that is a component of South Africa’s national health laboratory services.

**Methods:**

The study design was qualitative, exploratory, descriptive and contextual. Ten medical technologists were chosen using a purposive sampling technique. The data were gathered via in-depth, semi-structured interviews, which were audio recorded, professionally transcribed and coded. Tesch’s thematic coding method was used to perform a descriptive analysis.

**Results:**

Three key themes were identified: health and safety, negative experiences, and positive self-satisfaction. Medical technologists were concerned about their safety and health while handling COVID-19 specimens because of the challenging work environment caused by the pandemic. This resulted in negative experiences, including stress and anxiety. Despite this, several individuals felt their contributions throughout the pandemic were notable.

**Conclusion:**

The COVID-19 pandemic required additional resources, expertise, and training for medical technologists to safely collect and analyse biological samples.

**Contribution:**

The study’s findings present an opportunity to develop work-related risk management and support plans for future pandemics.

## Introduction

A medical technologist’s duties include collecting and analysing medical specimens, such as blood and urine samples. Medical technologists performing laboratory analyses remain largely anonymous, and very few people are aware that the results of diagnostic tests influence more than 70% of medical decisions (Przybylski 2020). During the coronavirus disease 2019 (COVID-19) pandemic, medical technologists were among the most crucial healthcare workers (HCWs) being required to perform thousands of polymerase chain reaction (PCR) tests to confirm severe acute respiratory syndrome coronavirus type 2 (SARS-CoV-2) infections, which cause COVID-19 (Wolszczak-Biedrzycka, Bieńkowska & Maksymowicz 2022). Because they interact with patients when collecting biological specimens and subsequently analysing them, medical technologists were highly exposed, making them extremely susceptible to contracting the virus (Javier et al. 2021). Medical technologists have also had to quickly create and perform novel tests for SARS-CoV-2 without being aware of the associated risks and learn how to operate in the face of significant uncertainty brought on by the COVID-19 pandemic, much like all other health systems and HCWs (Goff et al. 2022). This is because, at the time of COVID-19 development, little was understood about the disease’s hazards. The laboratory staff, however, were informed of the available facts regarding the illness.

Prior to the COVID-19 pandemic, medical technologists in South Africa (SA) examined 1 453 277 specimens between April 2018 and April 2019 (National Health Laboratory Services [NHLS] 2019). This figure encompasses all laboratory tests conducted. However, as COVID-19 cases increased starting in April 2020, medical technologists noticed a rise in the number of medical specimens that needed to be collected and examined. According to the SA National Institute for Communicable Diseases (NICD 2020) COVID-19 testing summary report, 2 042 889 COVID-19 tests were performed in SA by NHLS medical technologists between March 2020 and October 30, 2020. SA ranked fifth globally in terms of COVID-19 infections as of July 2020, with the province of Gauteng being the major source of these infections (Lewis & Mulla 2020). Additionally, Wilson et al. (2022) used the Occupational Health and Safety Information System (OHASIS) as a surveillance reporting tool to conduct a study to characterise the distribution of COVID-19 cases among medical laboratory staff. They found that 46% of COVID-19 cases analysed by medical laboratory staff were in the first wave of the virus, and 40% were in the second wave.

Given the greater volume of laboratory samples that needed to be collected and evaluated, occupational exposure to COVID-19 was more likely (Hawkins 2020). The risk of COVID-19 infection for medical technologists was increased because of the high contagiousness of the SARS-CoV-2. There exists contention among medical technologists and other HCWs that the COVID-19 infection ought to be categorised as an occupational disease that arises from exposure to harmful agents in the workplace or is linked to the work tasks performed (Goff et al. 2022). For instance, there is research that has explored and discussed the effects of COVID-19-related workload increases, with a primary focus on the psychosocial and mental well-being of HCWs and other workers (De Matteis 2021; Nyashanu, Pfende & Ekpenyong 2020; Salazar et al. 2020; Shreffler, Petrey & Huecker 2020; Sim 2020). Furthermore, research on the real-world experiences of HCWs, in general, has revealed several concerns and difficulties that they face (faced) in the pandemic, including higher rates of burnout, increased absenteeism from work because of illness and intentions to quit in addition to poor job satisfaction (Shehadeh 2020).

Nonetheless, there are still few studies that concentrate on medical technologists, who were among the most crucial HCWs during the pandemic (Cano et al. 2023; Javier et al. 2022; Wolszczak-Biedrzycka et al. 2022). Cano et al. (2023) looked at perceived job satisfaction of Filipino medical technologists during the COVID-19 pandemic, and based on their findings, they made recommendations for the development of staff retention programmes. In a study conducted in the Philippines by Javier et al. (2022), the day-to-day challenges that medical technologists assigned to COVID-19 specimen collection faced were qualitatively described. They found that these challenges can be classified into four categories: patient-related (such as attitude and health status), physical (such as fatigue), environmental (such as exposure to the virus), human and material resource-related and psychological (such as fear). By contrast, Wolszczak-Biedrzycka et al. (2022) examined the issues and concerns medical technologists in Poland had at the start of the pandemic, as well as how their attitudes changed over the course of the COVID-19 waves. They discovered that by the fourth wave, medical technologists had become accustomed to their work environment and the challenges posed. They did, however, stress the significance of having mental health support programmes aimed at medical technologists and all the other HCWs.

### Problem statement

Medical technologists, like any worker population, spend a substantial amount of time in the workplace, which makes it imperative that the workplace is safe and healthy to minimise the risk of acquiring occupational infections (ILO 2020; Department of Employment and Labour 2004). According to Matuka and Singh (2017), workplaces should have an infection control plan as an integral part of the occupational health and safety programme aligned to the OHSA. Despite all the precautionary measures, evidence is available confirming that occupational diseases do occur unexpectedly in the workplace (Department of Labour and Employment 2004), and enough literature is needed about medical technologists and their experiences of occupational exposure.

Only one study on medical laboratory services employees, which includes medical technologists, was conducted in SA (Wilson et al. 2022). The primary focus of the Wilson et al. (2022) study, which employed quantitative research design, was the COVID-19 cases among these medical laboratory services employees. This dearth of SA literature addressing subgroups of health workers, such as medical technologists, who may have had different physical, psychological and professional exposure to and preparation for COVID-19 disease because of the uniqueness of the tasks associated with their work of handling human specimens, served as the impetus for our study. The uniqueness of the hazards associated with the work of a handler of human specimens and the experiences of this occupational exposure during the COVID-19 pandemic could lead to targeted interventions for ensuring the safety, health and well-being of the medical technologists. This forms the basis for believing that the proposed study would contribute to the solution of the problem.

### Aim of the study

The aim of the study was to explore the experiences of medical technologists regarding medical specimen handling and their occupational exposure risk during the COVID-19 pandemic. Additionally, the study also intended to make suggestions for how support programmes should be designed considering these experiences.

### Definition of key concepts

#### Medical technologist

Medical technologists – also known as medical laboratory scientists – are professionals in the medical field that analyse human specimens such as bodily fluids, excretions and different body swabs for the purposes of laboratory diagnosis, treatment and research (Obeta et al. 2019). In this study, a medical technologist referred to this individual, who worked in a laboratory in the South African province of Gauteng and performed tests to help with COVID-19 diagnosis.

#### Medical specimen

A sample drawn from patients to perform a diagnostic test may include blood, urine, tissue biopsies and even swabs. These samples, particularly those with a respiratory origin, from the lung or respiratory system, were associated with medical specimens in this study.

## Research methods and design

### Research design

Medical technologists’ subjective experiences in our study were explored using a qualitative, exploratory, descriptive, contextual research design. This design was suitable for this research study, as medical technologists gave a rich description of their experiences (Gray, Grove & Sutherland 2019) of handling medical specimens during the COVID-19 pandemic which assisted the researcher in better understanding the experiences related to occupational exposure during specimen handling as recommended by Gray, Grove and Sutherland (2017:62). A qualitative research design adopts a person-centred and holistic perspective. It develops an understanding of people’s opinions and experiences of a specific topic (Christensen, Johnson & Turner 2020). Exploratory research is research conducted to gain new insights, discover new ideas and increase knowledge of a phenomenon (Bryman et al. 2017). Descriptive research focuses on describing a situation (Christensen et al. 2020). Contextual research refers to going out of a natural environment to observe behaviour and ask questions to the participants to explore their experiences (Malpass 2018).

### Research method

The study followed the guidelines of a phenomenology research approach as it focused on comprehending the medical technologist’s thoughts and perceptions of medical handling and occupational exposure during the pandemic in order to shed light on their unique experiences.

This research project’s approach relied on descriptive and exploratory research methods, which were adopted to describe and gain an understanding of these experiences of medical technologists. These made it possible to describe and explore the knowledge of the medical technologists, which goes beyond their training and work backgrounds but encompasses their viewpoints, interpersonal identities and experiences handling medical specimens and being exposed to occupational risk during the COVID-19 pandemic (Gray & Grove 2021).

### Setting

This study took place at a laboratory in the Gauteng province of South Africa. At the time of the study, the laboratory employed medical technologists who handled and analysed medical specimens and whose work extended to handling COVID-19 specimens and providing a COVID-19 diagnosis during the pandemic. Furthermore, the laboratory was chosen because it was situated close to the place of residence of the researcher.

### Study population and recruitment

The study’s target population consisted of all medical technologists, aged 18 years – 65 years, who worked in laboratories in Gauteng. Medical technologists employed at one of these Gauteng laboratories made up the accessible or sampling population. Ten medical technologists were chosen for this study using a purposive sampling technique based on several factors, such as having at least a year of experience handling medical specimens, registration with the health professions regulatory council (HPCSA), willingness to participate in the research and ability to communicate in English. Data collection continued until no new data emerged (data saturation) (Vasileiou et al. 2018). Convenience sampling for selecting the laboratory was deemed appropriate because of the laboratory being close to where the researcher lives, as recommended by Leavy (2017).

### Data collection

The study was conducted over two months (April 2022 – May 2022). Individual face-to-face, audio-recorded in-depth interviews, conducted by the researcher, with one opening open-ended question and probes pertaining to significant areas of interest were used to collect data. For this purpose, a private room available at the laboratory was used for the interviews as it was most convenient for the participants and also ensured privacy during interviews. For minimum to no disruptions during the interview process, a note was put on the door to indicate that a private interview session was in progress. The duration of each interview was approximately 45 min–60 min. Achieving data saturation (Saunders et al. 2018) was estimated to require a minimum of 10 interviews, given the study’s narrow focus and the medical technologists’ similar work environments. Answers to a single question, ‘*How was it for you working with medical specimens during the COVID-19 pandemic?*’ were collected along with demographic data, and the responses were subjected to thematic analysis.

### Data analysis

After reading the medical technologists’ responses several times to get a general idea of the content, the data were coded and categorised, and a central theme, themes and subthemes were created using Tesch’s thematic coding method for descriptive analysis (Creswell & Creswell 2018). Creswell and Creswell (2018) defined a central theme as the primary concept that recurs frequently throughout the field notes and audio-recorded interviews that have been transcribed. Tesch’s eight steps for data analysis were used, which included organising the vast amounts of information from the field notes and transcriptions to find related topics to group together and assigning codes, getting to know the participants’ spoken words by reading the transcriptions and listening to the audio recordings, constructing related topics that were then grouped together, establishing the most descriptive wording for the topics and categorising them. A final judgement about each category and its associated codes was also made, and links between the categories were developed. Quotations that provided support were gathered in a single place (Tesch 1990). An independent coder was recruited to perform an independent analysis following Tesch’s proposed steps. This enhanced the coding frame’s transparency and rigour when applied to the data collected, transcribed and analysed in this study (Cofie Braund & Dalgarno 2022).

### Trustworthiness

The Lincoln and Guba (1985) model, which comprises credibility, transferability, dependability and confirmability, was used to develop metrics for trustworthiness (Bryman et al. 2017). Through the use of purposeful sampling, we were able to locate participants from a variety of backgrounds who satisfied the inclusion requirements, giving us a range of perspectives on the occurrences seen (Moser & Korstjens 2018). This contributed to the credibility and authenticity of our results. Furthermore, by ensuring that the analysis went beyond the scope and subjectivity of a single individual, data saturation – until no new data arose during the interview process – and the participation of an independent coder enhanced the study’s analysis credibility (Cofie et al. 2022). Member checking, where medical technologists who took part in the study and where the supervisors of the study were given themes to go over to see whether they accurately represented their experiences, also ensured credibility.

Throughout the course of the research, the researcher who collected the data recorded her own experiences, observations and insights in a reflexive diary. The documentation of field notes, a thorough explanation of the findings and the inclusion of participant quotes all contribute to the study’s dependability and transferability (Polit & Beck 2017). Peer review briefing, independent coding and audio recording all contributed to the study’s confirmability. Peer review debriefing helped the researcher understand the phenomenon better and enhance the research inquiry by exposing the researcher to different points of view from multiple sources (Polit & Beck 2017).

### Ethical considerations

The study was approved by the Human Research Ethical Committee (HREC) at the University of Johannesburg (clearance no: REC – 828 – 2020). Participants received a letter of invitation prior to data collection, along with an information sheet outlining the purpose and goals of the study and the researchers’ email address. A consent form was sent to those who expressed interest in participating, and it was later reviewed with the participants when the interviews began. The assurance that their data would be kept private and confidential, along with their right to withdraw from the study at any time without facing penalties, was highlighted. Before the independent coder could access the participant data, they had to sign a confidentiality agreement contract.

## Results

The study participants’ demographic characteristics are presented in Table 1. Ten medical technologists were interviewed; eight of them identified as female, nine of them were unmarried and most of them had a National Diploma in Biomedical Technology, with an average duration of 4.1 years’ (ranging from 1 to 8 years) experience in medical specimen handling. Table 2 presents these themes and subthemes, which are further elaborated upon below, along with quotations that support the argument. After each quotation, there are brackets indicating the participant (P), their gender and age.

**TABLE 1:** Participant demographic characteristics.

Variables	P01	P02	P03	P04	P05	P06	P07	P08	P09	P10
Age (years)	29	27	31	28	28	26	34	29	28	28
Gender	Female	Female	Female	Male	Female	Female	Female	Female	Male	Female
Marital Status	Single	Single	Single	Single	Single	Single	Married	Single	Single	Single
Highest Qualification	National Diploma in Biomedical Technology	B. Tech in Biomedical Technology	National Diploma in Biomedical Technology	B. Tech in Biomedical Technology	B. Tech in Biomedical Technology	National Diploma in Biomedical Technology	National Diploma in Biomedical Technology	B Tech in Biomedical Technology	National Diploma in Biomedical Technology	National Diploma in Biomedical Technology

P, participant.

**TABLE 2:** Derived study themes and subthemes.

Themes	Subthemes
Occupational health and safety	1.1 Insufficient knowledge. 1.2 Organisational or environmental capacities. 1.3 Inadequate resources.
Negative experiences	2.1 Stress, frustration, depression and anxiety. 2.2 Role confusion and ambiguity. 2.3 Concern about heightened risk of transmission to family. 2.4 Isolation and societal stigmatisation.
Positive self-satisfaction	3.1 Adaptability and resilience. 3.2 Vigilance, learning and unlearning. 3.3 Societal applaud. 3.4 Community acceptance of the new normal.

Note: *Central theme*: Uncertainty regarding the safe handling of confirmed and suspected COVID-19 specimens, as well as the requirement to perform well in a mentally and physically demanding workplace.

### Central theme: Ambiguity on how to handle specimens of coronavirus disease 2019 and an expectation to perform in an emotionally and physically challenging working environment

According to the findings, all the participants agreed that the pandemic created a physically and emotionally taxing environment that increased their risk of becoming ill while handling medical specimens and of infecting their family members, which further contributed to their perception of being overexposed to the disease. At the start of the pandemic in 2020, when there were many health and safety issues, the participants admitted that this kind of setting made them feel more uncertain about how to manage medical specimens of proven or suspected COVID-19. This ultimately led to a variety of psychological stressors and anxiety, as well as dissatisfaction, role uncertainty and unhappiness.

Health and safety challenges of handling specimens at the beginning and during the COVID-19 pandemic were identified as the main sources of uncertainty and negative experiences by the participants. The following assertion corroborates this:

‘The fact that everything happened so quickly, no one knew the safest ways of handling the virus, the information was so limited, no one knew whether it was airborne or not and even the type of masks we had to wear. However, the specimens with COVID-19 were still brought in the laboratory, without us be provided with the necessary or relevant PPE set like masks, face shields, etc.’ (P09, male, 28 years old)

‘“I was surprised because we continued working with those questions. It was scary, I was not happy, we did not know what to anticipate so it was scary. I felt like we were dispensable because we were expected to do the job even though we had no information about the virus.” Looked upset and frustrated.’ (P09, male, 28 years old)

#### Theme 1: Occupational health and safety

Medical technologists found it more challenging to handle medical specimens during the pandemic because it required greater experience, training, knowledge of the virus and high-quality equipment to prevent infection. These issues, coupled with a rise in workload and a dearth of resources, added to the stressful work environment that every medical technologist confronted. Technologists who were interviewed highlighted the little amount of time they had to get ready for the crisis and the scarcity of information accessible in an already demanding work setting. Some of the following quotes sum up these experiences:

‘I knew that I have signed a contract to work but the fact that we were given COVID-19 specimens to handle, even though there was little information about the virus, made me feel we were dispensable. “There was not even a procedure to follow if I have to be exposed to the positive specimen”.’ (P09, male, 28 years old)

Some of the participants had to receive treatment for mental and physical issues because of the physically and mentally demanding work environment. This was because of the increased workload. The following assertions corroborate this:

‘I was admitted to the hospital with depression because I could not cope. I had to drop out of school. I could not study, my mind was not working well at that time. I was processing too many thoughts at the same time, I had too much breakdown. I went to see my therapist; he referred me to the psychiatrist. Part of my depression was because of work [*crying*].’ (P10, female, 28 years old)

‘We were put under a lot of strain and pressure because the samples were too many; you find yourself receiving more than 800 specimens per night. With the repetitive motion on your hands, the hands started becoming painful and the back becomes painful as well.’ (P10, female, 28 years old)

In addition, the degree of occupational exposure at work, whether real or perceived, worried the participants. The most frequently cited factor as an example of risk exposure was the lack of personal protective equipment (PPE) needed to perform one’s job:

‘When you look at the safety part it was lacking, we were not protected. We were given three gloves to use in 12 hours, you cannot even go to the toilet because that means I must change the gloves.’ (P10, female, 28 years old)

‘We work at night and there is no sanitiser, but we are expected to process samples. I felt like I need to properly sanitise and wipe my hands.’ (P10, female, 28 years old)

‘We did not have a health and safety nurse in the laboratory, but since the pandemic was brought, she assists us with counselling and guidance. In general, the health and safety team played an important role. Having a nurse in the laboratory made us feel much better.’ (P04, male, 28 years old)

#### Theme 2: Negative experiences

Medical technologists had a negative experience handling medical specimens during the COVID-19 pandemic. This was mostly caused by self-reported ignorance stemming from a lack of knowledge about COVID-19, doubt about the virus and conflicting information about the disease from the media, government regulations and general data. The uncertainty surrounding the coronavirus illness led to images of fear of the unknown, frustration and worry. According to one participant:

‘The receiving of the specimens is done by the laboratory support service then you will not know which specimen has suspicion of COVID-19, which made things difficult.’ (P07, female, 34 years old)

Another participant echoed the anxiety of not knowing what to expect in terms of potential coronavirus infection:

‘“Mmm, Yeah! It was scary because it was a new thing, you will never know when you will get infected, and might even get infected when handling the specimen”. Looking frustrated.’ (P05, female, 28 years old).

Expressions of fear about bringing the illness home and responses from the community, particularly rejection, also revealed the participants’ anxiety. One participant confirmed the other participants’ concern about infecting family members, saying:

‘“Firstly, I feared what if I get the virus and bring it home, at the time my son was four, and my mother is old”. “It was just stressful because you ask yourself, who do I bring the infection to, will I bring the infection home and kill someone, what do I do, do I have to social distance from the baby as well, who is always all over me?”.’ (P01, female, 29 years old)

Certain participants expressed that they were subjected to stigma from the community because of their place of employment:

‘I feel like there was a stigma, people thinking because you work at the hospital you have COVID-19.’ (P02, female, 27 years old)

‘The community was scared to be closer to me and thought we might have a COVID-19 infection. There was some kind of stigma.’ (P08, female, 29 years old)

Because of the stigma and rejection from the community, the medical technologists experienced a unique kind of social isolation in which they were forced to actively cut off contact with their family members. As one participant puts it:

‘She [*mother*] was scared for me, there was a time when I could not go home as much as I would have liked to because I was scared of what if I have COVID-19, especially since we were not tested often for COVID-19.’ (P03, female, 31 years old)

#### Theme 3: Positive self-satisfaction

The participants conveyed that there were good aspects to their experiences handling specimens during the COVID-19 pandemic. Most participants clarified that while handling specimens was initially unclear and perplexing, it did, in the end, result in some beneficial experiences. For example, as new information about the coronavirus disease surfaced daily, it made it possible to provide support for the workplace environment by redrafting safety operating procedures for handling medical specimens that may contain COVID-19, having visible leadership, holding intensive information sessions and offering effective personal protective clothing and equipment. These structures subsequently served as models for specific compliance behaviours pertaining to COVID-19 safety. The statements that follow made this clear:

‘We were worried, we were thinking of death. This was because there was a lack of information, we were not sure of so many things but now is better because there is more information.’ (P07, female, 34 years old)

‘I realised that I’m safer here at work than outside …’ (P01, female, 29 years old)

‘My manager provided moral support because the first day she worked with us the whole night.’ (P10, female, 28 years old)

A few medical technologists mentioned feeling a deep sense of satisfaction, knowing that even in hard circumstances, they were making a big difference for society. This inner sensation was further reinforced by the community’s shift in perspective after originally rejecting them.

The following claims substantiate this:

‘As much as it was scary and overwhelming, but there was that feeling that you are playing an important role and making a difference in people’s lives, so that motivated me and gave me courage.’ (Participant 05, female, 28 years old)

‘The community [*now*] knew better about us, so they will even give you advice.’ (P07, female, 34 years old)

## Discussion

To effectively monitor and oversee COVID-19, the World Health Organization (WHO 2020) emphasised the importance of ‘detect, protect, and treat’ to halt the spread of SARS-CoV-2 and COVID-19. Prompt medical laboratory testing and treatment thus significantly decreased the number of COVID-19 cases. This necessitated testing of the asymptomatic and seemingly healthy population in addition to suspected cases or contacts (Obeta et al. 2020). Medical technologists may have had a legitimate fear of being at high risk of contracting the disease because of the subsequent surge capacity to process a large volume of specimens, pressure to confirm patient diagnoses, frequency and level of exposure and the virulent nature and impact of the disease. According to Qiu et al. (2021), self-risk perception has an intermediary function in both the employee mental health and the experience of the severity of the disease. According to some participant accounts, these conditions of high workloads, which were exacerbated by organisational shortcomings and ambiguous procedures, led to mental health issues, which manifested as stress, anxiety, depression and emotional exhaustion (Qiu et al. 2021; Pappa et al. 2020). Penninx et al. (2022) and Wu et al. (2021) note that although there was a slight rise in self-reported mental health issues during the COVID-19 pandemic, this has resulted in higher objectively quantifiable incidence of mental illnesses, self-harm or suicide among the public. This may indicate less effective adaptation and resilience.

According to this research study’s reports, medical technologists’ two main concerns were returning the virus to their families (Urooj et al. 2020) and the stigma that the public had against healthcare professionals who were thought to be carriers of the disease (Kelly et al. 2020; Pappa et al. 2020). These disease transmission-related worries highlight the need for efficient infection control procedures as methods to guarantee health and safety and dispel anxiety (Urooj et al. 2020). Initially, the medical technologists did not feel safe; only over time, they did feel safe when a ‘routine’ for dealing with COVID-19 specimens was established. The report from the workers was not unexpected considering the following aspects of the workplace: A regulated environment; legal frameworks pertaining to occupational health and safety that require employers to take reasonable precautions; the creation of structures and resources to support health initiatives, including occupational health services and policies and the fact that workers spend a larger percentage of their waking hours at work. Balkhi et al. (2020), Taylor et al. (2020) and Usher, Bhullar & Jackson (2020) confirm that stigmatisation and social isolation of medical personnel from their family members increased psychological distress such as frustration, loneliness and anxiety. According to Bagcchi (2020), medical personnel faced challenges such as being denied access to public transportation in various parts of the world, including Mexico, India, Zimbabwe and Malawi. Some of them even faced eviction from their rented apartments and physical abuse in public. These events compounded the already difficult circumstances faced by them. Medical technologists had to deal with their families’ reactions on top of the stigma they faced from the communities. As human adaptability and resilience are closely associated with the extent and quality of interpersonal relationships, including community and group participation (Pietrabissa & Simpson 2020), it became imperative that targeted interventions, such as dispelling societal and communal stigma through accurate and clear information on COVID-19, be controlled.

The COVID-19 pandemic-related novel protocols and the normal pre-pandemic protocols created in medical laboratories clearly differed in their operational aspects. The adoption of these new work procedures and guidelines in situations where there was a lack of skilled resources or a need for additional training, inadequate or lack of personal protection, as well as poor knowledge and conflicting messages about the disease that were powered by uncontrolled, free-flowing information (Murugan, Rajavel & Singh 2021) led to medical technologists being much more confused and uncertain. The participants in this study had an average of just above 4 years of experience in handling medical specimens. It may therefore not be surprising that role confusion and ambiguity on how to handle medical specimens, which potentially contained COVID-19 emerged as a key issue in the study. The challenges that medical technologists faced during the COVID-19 pandemic were not limited to the absence of defined strategies or guidelines for diagnostic treatments. The participant experiences also disclosed other noteworthy challenges that are in line with those documented in the literature: challenges relating to resources, such as the lack of human resources with the necessary training (Cano & Olano 2022; Uchejeso et al. 2021); environmental issues, such as the need to handle large volumes of specimens and the lack of PPE in conjunction with inadequate training (Karlsson & Fraenkel 2020) and psychological issues. These factors combined to create a physically and emotionally taxing work environment. (Wu et al. 2021)

Despite these negative experiences, there were some positive experiences that the medical technologists could share, even though they seemed to have surfaced as knowledge about the coronavirus disease improved. These experiences included social support from coworkers, a sense of community acceptance, visible leadership and an increased sense of satisfaction that work management had given them reasonably practical measures to protect them from COVID-19 (Jafri, Ahmed & Siddiqui 2020; Willis et al. 2021). Over time, this became evident in the participants that when workers use their social, personal and professional networks to adapt swiftly to change and uncertainty at work, they are exhibiting resilient behaviour (Ojo, Fawehinmi & Yusliza 2021).

Medical technologists are a comparatively small subset of medical professionals that have mostly received little attention from the public. In most of the literature on lived experiences of health professionals during the COVID-19 pandemic, doctors and nurses have been the primary subjects. This study’s primary strength is that it makes one of the first attempts to explore the experiences of this small group of medical professionals in the South African context during the COVID-19 pandemic and describes how their total health and well-being is related to actual or perceived occupational exposure in their field of work. The findings, conclusions and recommendations of the study may be cautiously transferable to all other medical professionals.

### Limitations of the study

As this research study’s report concentrates on a limited sample of 10 technologists who were recruited from a single laboratory in a single province, the study findings are contextual and therefore are not necessarily indicative of the experiences of these medical technologists in a larger context. Transferring the findings to other medical professionals requires caution. Greater understanding of the phenomenon that may not have been possible in this study could be obtained with a bigger sample size. Future research is possible in any of these areas.

### Recommendations

The findings of this study allow for the formulation of the following recommendations to enhance the performance of medical laboratory scientists during preparation for future pandemics.

Firstly, the ability of the medical technologists to develop resilience after their negative experiences of handling medical specimens and a reported high self-perception risk of occupational exposure was observed as being influenced by support from the employer and occupational medical practitioners in various ways. When faced with a pandemic like COVID-19, occupational health specialists should initiate a formal issue-based risk management survey swiftly. This will help the employer or organisation identify potential risks, evaluate resources and suggest the most effective ways to limit exposure to the illness and its spread.

Secondly, this study found that information flowed freely and uncontrolled, creating contradictory messages. Additionally, it is recommended that the employer take greater control over the narrative around occupational exposure by giving explicit instructions on how to handle medical specimens in the event of the COVID-19 pandemic. In accordance with *Section 8 of the OHS Act* (Department of Employment and Labour 2004), one of the employers’ responsibilities is to give the required guidance, instruction and oversight while taking the employee’s level of competency into consideration.

Thirdly, assertions of increased mental health problems, such as stress, anxiety, depression and fear, that are exacerbated by isolation with little indication about how companies and employees addressed these problems indicates the need for an all-encompassing strategy that takes into account the organisational and social causes that led to these problems and includes the acquisition of a social worker or mental health service to address the psychosocial well-being of the medical laboratory employees.

## Conclusion

This study explored medical technologists’ experiences when handling medical specimens during the COVID-19 pandemic. It found a range of experiences from the pre-pandemic period to the initial COVID-19 surge and during the pandemic. The challenges that were encountered and the lessons learned during these times indicate that certain aspects of occupational health and safety may have been handled better. The challenges that the medical technologists reported to have faced were directly linked to their unfavourable experiences (role confusion, frustration and stress, societal stigmatisation and isolation). These challenges placed greater mental strain on these frontline workers who were already understaffed and working in a healthcare system that was already overburdened.

Because it was crucial to restore both individual and overall equilibrium, medical technologists made every effort to identify and address these disruptions when they could. The medical technologists were generally socially isolated but appreciated the role that family members as a support system could have played in the process. Positive experiences are then generated by the medical technicians’ progressive development of resilience and adaptability. Workplace occupational health and safety policies and the application of preventative measures made this resilience and adaptation possible. This was also considered to be the outcome of numerous supporting structures derived from various social interactions or connections.
